# Marsdenia tenacissima enhances immune response of tumor infiltrating T lymphocytes to colorectal cancer

**DOI:** 10.3389/fimmu.2023.1238694

**Published:** 2023-08-15

**Authors:** Ben Yi, Shuai Zhang, Suying Yan, Yanfei Liu, Zhiqiang Feng, Tianhao Chu, Jun Liu, Wei Wang, Jun Xue, Chunze Zhang, Yijia Wang

**Affiliations:** ^1^ School of Integrative Medicine, Tianjin University of Traditional Chinese Medicine, Tianjin, China; ^2^ Department of Colorectal Surgery, Tianjin Union Medical Center, Tianjin, China; ^3^ Department of Radiology, The Fourth Central Hospital Affiliated to Nankai University, Tianjin, China; ^4^ TEDA Institute of Biological Sciences and Biotechnology, Nankai University, Tianjin, China; ^5^ Department of General Surgery, The First Affiliated Hospital of Hebei North University, Zhangjiakou, China; ^6^ Laboratory of Oncologic Molecular Medicine, Tianjin Union Medical Center, Tianjin, China

**Keywords:** marsdenia tenacissima extraction, tumor microenvironment, tumor infiltrating T cells, CD8, colorectal cancer

## Abstract

**Introduction:**

Tumor-infiltrating T lymphocytes in the tumor microenvironment are critical factors influencing the prognosis and chemotherapy outcomes. As a Chinese herbal medicine, Marsdenia tenacissima extract (MTE) has been widely used to treat cancer in China. Its immunoregulatory effects on tumor-associated macrophages is well known, but whether it regulates tumor-infiltrating T-cell functions remains unclear.

**Method:**

We collected 17 tumor samples from MTE-administered colorectal cancer patients, 13 of which showed upregulation of CD3+/CD8+ tumor-infiltrating T cells. Further *in vitro* and *in vivo* experiments were performed to investigate the regulatory effects of MTE on tumor-infiltrating T cells and immune escape of tumors.

**Results:**

Under single and co-culture conditions, MTE inhibited TGF-β1 and PD-L1 expression in the colorectal cancer (CRC) cell lines HCT116 and LoVo. In Jurkat cells, MTE inhibited FOXP3 and IL-10 expression, increased IL-2 expression, but had no effect on PD-1 expression. These findings were confirmed *in vitro* using subcutaneous and colitis-associated CRC mouse models. MTE also increased the density of CD3+/CD8+ tumor-infiltrating T cells and exhibited considerable tumor-suppressive effects in these two tumor mouse models.

**Conclusions:**

Our findings suggested that MTE inhibits the immune escape of cancer cells, a precipitating factor increasing the immune response of T lymphocytes.

## 1 Introduction

Colorectal cancer (CRC) is a common malignant neoplasm that is suitable for surgical excision in its early stages. However, recurrence still occurs in some patients, even after surgery and chemotherapy. For example, the docetaxel-5FU-oxaliplatin FLOT regimen is the standard perioperative treatment for CRC; however, its curative effect is limited by resistance (1). Tumor microenvironment plays a key role in the development of tumor resistance to the immune system and anti-tumor drugs. Patient immunity is a determining factor in the tumor microenvironment; therefore, immune regulation via adjuvant drugs improves prognosis (2). Tumor-infiltrating lymphocytes such as regulatory T cells (Tregs), tumor-associated macrophages (TAM), and cytotoxic T cells are important components of the tumor microenvironment. The immunoscore, which is the total count of CD3+ tumor-infiltrating T cells and CD8+ cytotoxic tumor-infiltrating T cells, is associated with the prognosis of CRC patients (3). Patients with high densities of CD3+/CD8+ tumor-infiltrating T cells have the lowest risk of recurrence at five years (3). Therefore, the regulation of anti-tumor drugs in tumor TME should be considered perioperatively.

Marsdenia tenacissima (Roxb.) Wight and Arn (MT), a member of the Asclepiadaceae family mainly produced in Yunnan (China), is a well-known traditional Chinese medicine used for cancer treatment. MT directly suppresses tumor growth through inhibition of the MIF/mTOR signaling to induce autophagy and apoptosis in hepatocellular cells (4). It also enhances the effects of other chemotherapeutic drugs. MT has been reported to reverse the multidrug resistance of cervical carcinoma cells to paclitaxel by inhibiting P-gp and MRP2 in cancer cells (5).

Marsdenia tenacissima extract (MTE), derived from MT, is widely used to treat cancer in China. As an adjuvant, MTE increases the anti-tumor activity of many chemotherapeutic drugs. For example, MTE combined with cisplatin significantly reduced the invasion and migration of ovarian cancer cells (6). It enhances paclitaxel efficacy in ovarian cancer by suppressing the expression of the pregnane X receptor and its downstream molecules (7). MTE also has an immune regulatory effect on the tumor microenvironment. It has been reported to enhance macrophage polarization from M2 to M1 phenotype through HDGF inhibition in non-small cell lung cancer (8). However, research on the function of tumor-infiltrating T cells in the TME remains lacking.

We evaluated the immunoregulatory effect of MTE on tumor-infiltrating T cells in CRC, using tumor tissues of patients before and after MTE treatment. Based on the preliminary findings, *in vitro* and *in vivo* experiments were performed. A co-culture system of CRC and Jurkat T cells was used to investigate the influence of MTE on the interaction between tumor and T cells *in vitro*. To study the effects of MTE *in vivo*, we used a colitis-associated CRC model induced by azoxymethane (AOM)/dextran sodium sulfate (DSS) and a CT26 cell subcutaneous tumor model in BALB/c mice. Our research innovatively focused on the immunoregulatory effects of MTE on tumor-infiltrating T cells in CRC, providing a potentially reliable theory for the improvement of prognosis by MTE treatment.

## 2 Materials and methods

### 2.1 Reagents and antibodies

Marsdenia tenacissima tablet (#Z20064221) were purchased from Suzhong Pharmaceutical Co. Ltd (Taizhou, Jiangsu, China). Marsdenia tenacissima injection (#Z20025868) were purchased from Nanjing Sanhome Pharmaceutical Co. Ltd (Nanjing, Jiangsu, China). The marsdenia tenacissima tablet is suitable for oral administration, but it is inconvenient for experimental research. The tablet and injection have the same active constituent, several forms of Tenacissosides. Human XL Cytokine Array Kit (#ARY022B) was purchased from R&D Systems, Inc (Minneapolis, MN, USA). Rabbit anti-CD3 antibody (#85061), rabbit anti-CD8 antibody (#85336), rabbit anti-PD-1 antibody (#86163) and rabbit anti-PD-L1 antibody (#13684) were purchased from Cell Signaling Technology Co., Ltd (Danvers, MA, US). Goat anti-rabbit IgG-HRP antibody (#P0615) were purchased from Beyotime Biotech Inc. Rabbit anti-FOXP3 antibody (#PB0043) was purchased from Boster Biological Technology Co., Ltd (Wuhan, Hubei, China). Rabbit anti-GAPDH antibody (#A19056) was purchased from ABclonal Technology Co.,Ltd (Wuhan, Hubei, China). Rabbit anti-TGF-β1 antibody (#abs130620), human IL-2 ELISA Kit (#abs510001), human IL-10 ELISA Kit (#abs510005), mouse IL-2 ELISA Kit (#abs520002), mouse IL-10 ELISA Kit (#abs520005), human/mouse TGF-β1 ELISA Kit (#abs552208) were purchase from Absin Biotechnology Co., Ltd (Shanghai, China). AOM was purchased from Aladdin Biochemical Technology Co.,Ltd (Shanghai, China). DSS was purchased from Shanghai yuanye Bio-Technology Co., Ltd (Shanghai, China). Phorbol 12-myristate 13-acetate (PMA) was purchased from Abcam Trading Co., Ltd (Shanghai, China). Ionomycin was purchased from Yeasen Biotechnology Co., Ltd (Shanghai, China). BCA protein quantification kit was purchased from Solarbio Science & Technology Co.,Ltd (Beijing, China). AST activity assay kit (#BC1565) and ALT (#BC1555) activity assay kit were purchased from Solarbio Life Sciences Co., Ltd (Beijing, China).

### 2.2 Cell lines

Human colon cancer cell lines HCT116 and LoVo, human acute leukemia cell line Jurkat T, murine colon cancer cell line CT26 were purchased from the Shanghai Institutes for Biological Sciences, Chinese Academy of Sciences (Shanghai, China). All cells were cultured in RPMI 1640 medium supplemented with 10% FBS, 100μg/ml streptomycin, and 100 U/ml penicillin. Jurkat T cells were treated by 10 ml complete medium contained 50 ng/ml PMA and 1 μg/ml ionomycin in 10 cm dish for 6 h to be activated. The activated Jurkat T cells were used in following experiments.

### 2.3 Patients

We collected tumor tissues and serum samples from 17 patients with stage II and III CRC, diagnosed by colonoscopy. The patients were given marsdenia tenacissima tablet (2.4 g) orally, thrice a day, for two weeks according to the physician’s order. Tumor tissues were collected by colonoscopy approximately three days before taking the tablet and by surgical operation one day after taking the tablet. Sera and tumor tissues were collected at the same time points. These patients had not received other anti-tumor drugs before sample collection.

### 2.4 Immunohistochemistry

Colorectal tumor tissues of patients, subcutaneous tumor tissues of mice and colorectal tissues of AOM/DSS treated mice were fixed in 10% buffered formalin. After 48 h, tissues were embedded in paraffin and prepared to slices with 4 μm. Paraffin sections were incubated in 10 mM sodium citrate solution (pH 6) after deparaffinization to retrieve antigen. The sections were then incubated with primary antibody (dilution 1:200) at 4°C overnight. And next the sections were washed and incubated with goat anti-rabbit IgG-HRP antibody (dilution 1:100) for 2 h at room temperature. The color was developed by DAB substrate kits and images were captured with magnification ×200. The positve stained cells were counted using the Image-pro plus software (Meyer Instruments, Inc.; TX, USA).

### 2.5 Measurement of cell viability

HCT116, LoVo and Jurkat T cells were treated with MTE injection to confirm suitable concentration of MTE in the following experiments. Briefly, cells were cultured in 96-well plates with a concentration of 5×10^3^ cells per well and treated with MTE for 48 h. CCK-8 solution was used to measure cell viability after MTE treatment. A microplate reader (Synergy HT; Bio-Tek, USA) was used to measure absorbance at the wavelength of 450nm.

### 2.6 Human XL cytokine array analysis

The Human XL Cytokine Array kit was used to determine the MTE-induced changes in various cytokines in Jurkat T cells. Briefly, the cells were treated with MTE for 48 h, and the supernatants were harvested and assayed for cytokines according to the manufacturer’s instructions. The levels of cytokines were quantified using the ImageJ software (National Institutes of Health, Bethesda, MD, USA).

### 2.7 Co-culture experiments

HCT116 or LoVo cells were cultured in 6-well plates at 1×10^6^/well. The cells were washed with PBS, and fresh culture medium was added. Activated Jurkat T cells (1×10^6^) were added to a transwell chamber (0.4 μm aperture), which was placed into the dishes. The indirect co-culture structure ensured the Jurkat T cells were not in contact with cancer cells. Cells and supernatants were harvested after 48 h.

### 2.8 Western blotting analysis

Cells were lysed on ice by RIPA buffer containing protease inhibitor cocktail and protein concentration was determined by BCA protein quantification kit. Protein were suspended in the SDS loading buffer and boiled for 10 min. Equal amounts (30 μg protein/line) of protein were resolved by a 8-10% SDS-PAGE gel and transferred onto Immobilon PVDF membranes which were then blocked with 5% skim milk. After that, the membranes were incubated with primary antibodies overnight at 4°C, and then were labeled with HRP-conjugated second antibodies for 1 h at room temperature. Immunoreactive bands were detected by incubating with HRP-conjugated goat anti-rabbit IgG secondary antibody (1:10000) and enhanced chemiluminescence reagent. The amount of the proteins was measured using ImageJ software and normalized to their respective control. Each assay was performed in triplicate.

### 2.9 Enzyme-Linked Immunosorbent Assay for TGF-β1, IL-2 and IL-10 detection

TGF-β1, IL-2 and IL-10 levels in the cell supernatants and patient or mice sera samples were detected using the human or mouse ELISA detection kit, according to manufacturer’s protocol.

### 2.10 Colitis associated CRC mouse model

A colitis-associated CRC (CAC) tumor model was established to evaluate the immunoregulatory effects of MTE on intestinal carcinoma in situ. Briefly, 4-5-week-old female Balb/c mice were purchased from Beijing Hfkbio Co., Ltd. (Beijing, China). AOM and DSS were dissolved in sterile water. AOM (10 mg/kg) was administered by intraperitoneal injection once, following which the mice drank water for seven days. Next 2.5% DSS solution was administered for seven days, followed by drinking water for 14 days, the first period of alternation between DSS and water. At the end of the fourth period, 5, 10 or 20 ml/kg MTE was administered intraperitoneally every alternate day. Only water was consumed for drinking purposes. The mice were sacrificed 14 days after dosing. The intestines from the ileocecal to the anus, liver, lungs, kidneys, spleen, and serum were collected. The length of the intestine and number of tumors were measured. Tissues were fixed in 10% buffered formalin for hematoxylin and eosin staining and immunohistochemistry. Serum samples were used for TGF-β1, IL-2, and IL-10 measurements.

### 2.11 Subcutaneous tumor mouse model

A subcutaneous tumor model was established to further evaluate the immunoregulatory effects of MTE *in vivo*. Female BALB/c mice (4-5 week-old) were purchased from Beijing Hfkbio Co., Ltd. (Beijing, China). CT26 cells (1×10^6^ in 200 ml) were injected subcutaneously into one side of the mouse armpit. Seven days later, MTE (5, 10 or 20 ml/kg) was administered intraperitoneally every other day. The mice were sacrificed 14 days after dosing. Tumor, liver, lung, kidney, spleen, and serum samples were collected. The tumor weights were measured. Tissues were fixed in 10% buffered formalin for hematoxylin and eosin staining and immunohistochemistry. Serum was used to determine TGF-β1, IL-2, IL-10, AST, and ALT levels.

### 2.12 Statistical analysis

All data are represented as the mean ± S.D. One-way ANOVA analysis of variance was used to evaluate statistical differences among multiple groups, student’s t test was used to evaluate statistical differences between two groups by the GraphPad software (GraphPad Inc., San diego, CA, USA). *P* < 0.05 represents statistical significance difference.

## 3 Results

### 3.1 MTE regulates tumor infiltrating T lymphocytes of CRC patients

**Figure 1:**
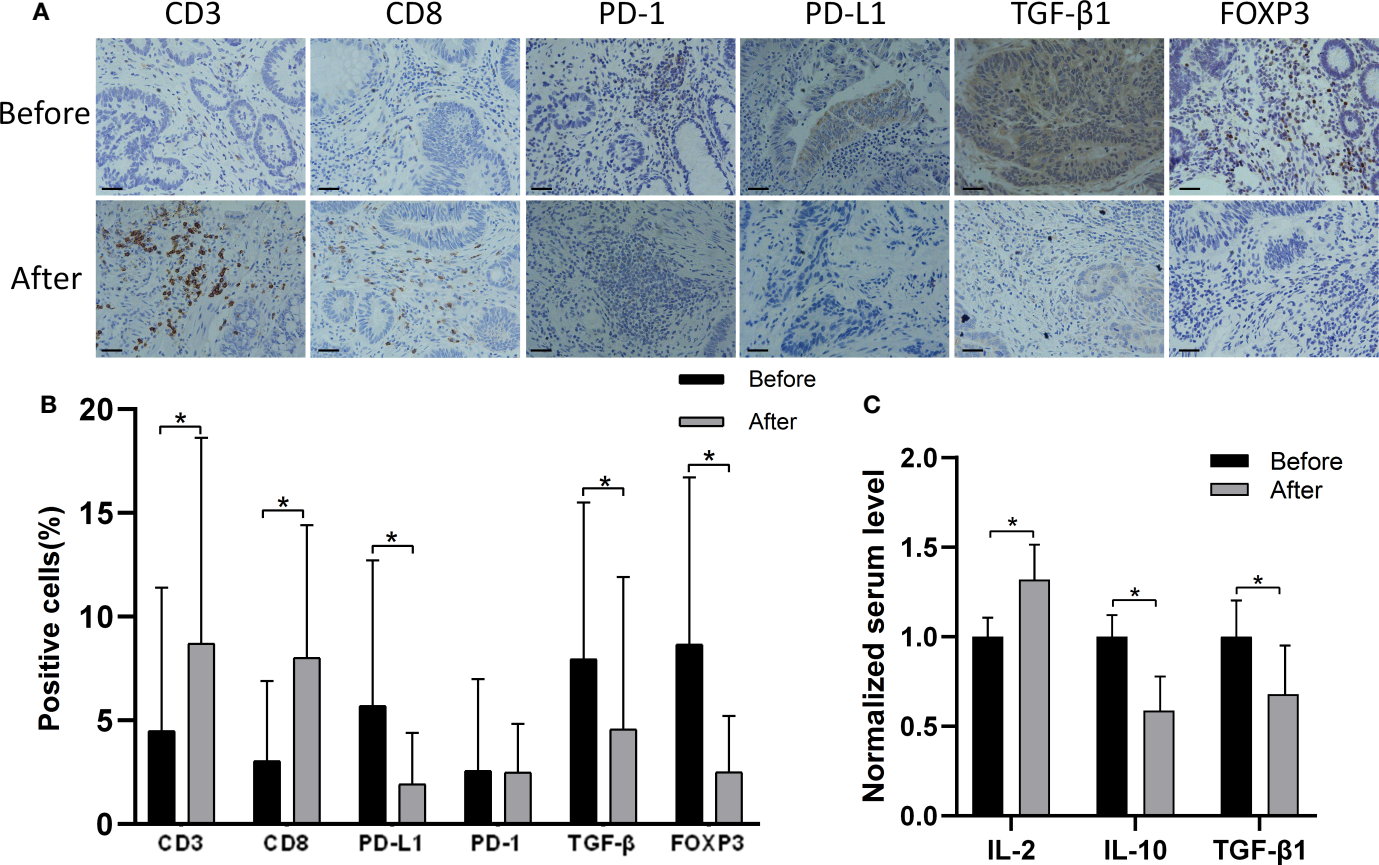
Immunohistochemistry findings from tissue samples of CRC patients before and after MTE treatment. **(A)** Shown are the findings from one patient. Results of other 16 patients are shown in **Figure S1**. **(B)** The bar graph compares the expression of PD-1, PD-L1, TGF-β1(tumor), and FOXP3 before and after MTE treatment in all 17 patients. **(C)** Serum levels of IL-2, IL-10 and TGF-β1 were compared before and after treatment in all 17 patients. Cytokine concentrations were normalized against the untreated group. *P < 0.05.

**Table 1** summarizes the findings from 17 patients with stage II/III CRC who were treated with MTE before surgery. Immunohistochemistry showed an increase in the number of CD3+/CD8+ tumor-infiltrating T lymphocytes in 13/17 patients, following MTE therapy (**Figure 1**), implying that MTE may improve prognosis through immunoregulation. Following MTE treatment, the PD-1 expression in tumor-infiltrating lymphocytes (TILs) was unchanged, but PD-L1decreased in the tumor cells in 9 patients, suggesting a decrease in the immune escape of cancer cells by PD-L1 inhibition. MTE treatment reduced TGF-β1 expression within the tumor cells and FOXP3 expression in the TILs. These results suggest that MTE inhibits T_regs_ differentiation by inhibiting TGF-β1 secretion in cancer cells. Analysis of serum samples showed that most of patients have increased IL-2, and decreased IL-10 and TGF-β1.

**Table 1:** Summary information for the colorectal cancer patients.

Information	CRC patients (17)	
	Infiltration increased	Infiltration unchanged
Rectum/Colon	8/5	2/2
Age (range/mean ± STD)	42-70/60±10	42-73/61±14
Stage (II/III)	5/8	2/2
PD-1	2/11	1/3
PD-L1	7/6	2/2
TGF-β1(tumor)	8/5	2/2
FOXP3	7/6	3/1
IL-2	12/1	4
IL-10	12/1	4
TGF-β1(serum)	9/4	2/2

‘Infiltration increased’ indicates an increase in the number of CD3+ and CD8+ tumor infiltrating T lymphocytes. ‘Infiltration unchanged’ indicates the number of at least one type T cells was unchanged. While one patient showed no change in the numbers of CD3+ and CD8+ T cells, none showed a decrease in both CD3+ or CD8+ T cells. The expression of PD-1, PD-L1, TGF-β1(tumor), and FOXP3, and serum levels of IL-2, IL-10, TGF-β1(serum) showed different trends following MTE treatment. Green represents decrease, red represents increase, while black represents no change after MTE treatment.

### 3.2 MTE regulates Jurkat cell function and differentiation

**Figure 2:**
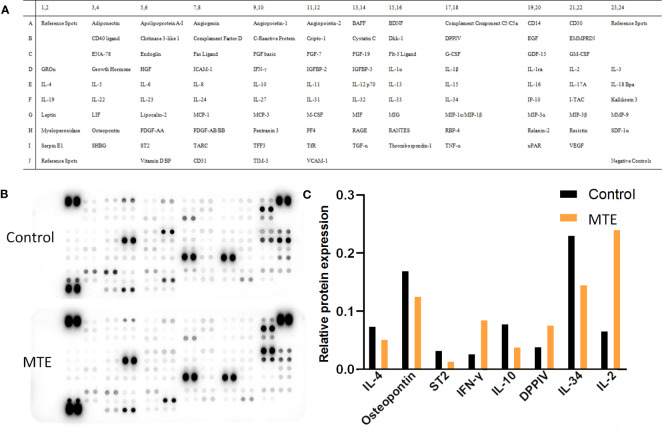
Human XL Cytokine Array of 105 proteins in MTE treated Jurkat T cells. **(A)** The array included multiple cytokines, chemokines, growth factors and other soluble proteins in the culture supernatants. Each protein was spotted in duplicate, and three pairs of positive controls were added in the three corners (top and bottom left and top right) and a pair of negative control in the bottom right corner. **(B)** Images of the exposed membranes. ‘Control’ represents untreated Jurkat cells. **(C)** Statistical results of some proteins which relate to function and differentiation of T cells are shown.

The Human XL Cytokine Array studies showed that MTE treatment regulated the levels of cytokines, some of which were related to the differentiation and immune function of T lymphocytes (**Figure 2A**). In general, MTE upregulated the immune function of Jurkat cells. Variations in some typical cytokines related to the tumor microenvironment are shown in **Figures 2B, C**. DPPIV is a T-cell activation marker expressed in CD8+ T cells, and its upregulation promotes IL-2 production (9). The latter is mainly produced by activated CD4+/CD8+ T cells and promotes the T cell response to tumors (10). MTE increased secretion of DDPIV, IL-2 and IFN-γ, promoting the immune response of TILs to cancer cells. The levels of anti-inflammatory cytokines, IL-4 and IL-10, decreased after MTE treatment. IL-10 plays an immunosuppressive role by reducing IL-2 secretion (11), is related to Treg cell differentiation (12) and induces M2 polarization of TAM (13). IL-34 caused an immunosuppressive microenvironment and increased TAM infiltration in CRC (14), which was inhibited by MTE. MTE inhibits osteopontin, which is elevated in human CRC, and may function as an immune checkpoint and potent T cell suppressor (15). MTE downregulates ST2, a factor that drives Tregs to accumulate in the tumor microenvironment (16), was down regulated by MTE.

### 3.3 MTE inhibits immune escape of CRC cells and regulates Jurkat T cell functions *in vitro*

**Figure 3:**
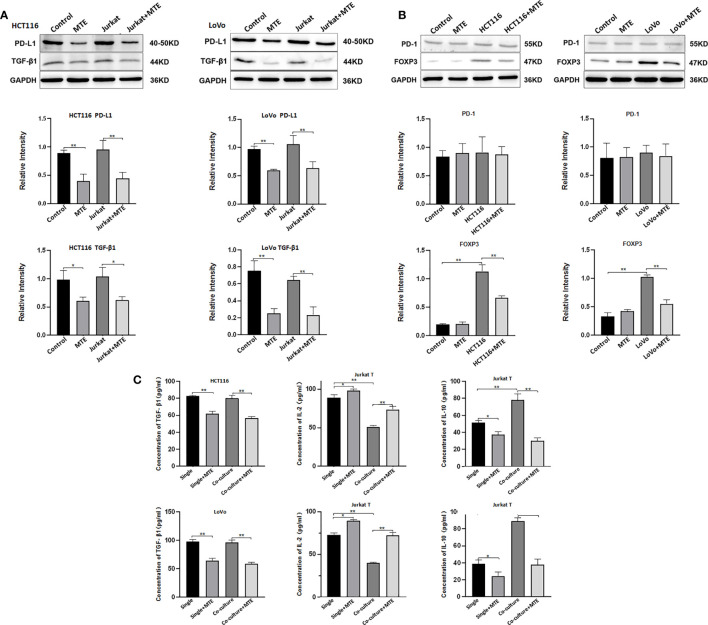
Western blotting and ELISA of HCT116, LoVo and Jurkat T cells treated with MTE treated in single culture or co-culture condition. **(A)** Western blot analysis of PD-L1, TGF-β1 expression in HCT116 or LoVo cells, single cultured or co-cultured with Jurkat T cells and treated with MTE, which were labeled in top row of blots or bottom row of column chart. **(B)** Western blot analysis of PD-1 and FOXP3 expression in Jurkat T cells single cultured or co-cultured with HCT116 or LoVo cells, and treated with MTE, which were labeled in top row of blots or bottom row of column chart. **(C)** ELISA analysis of supernatants from single cultures or co-cultures. In single culture, supernatants of HCT116 or LoVo cells was used to test TGF-β1 secretion, and supernatants of Jurkat T cells was used to test IL-2 and IL-10 secretion levels.*P < 0.05, **P < 0.01.

Based on the cell viability results shown in **Figure S2**, HCT116 and LoVo cells were treated with 12 mg/ml and 16 mg/ml of MTE, respectively in a single or co-culture system. The same concentration of MTE was used to treat Jurkat T cells co-cultured with the corresponding CRC cells. MTE treatment inhibited PD-L1 expression in HCT116 and LoVo cells (**Figure 3A**). Hence, as CRC cells, both HCT116 and LoVo have immune escape ability through PD-L1, and MTE treatment decreases it. Furthermore, both HCT116 and LoVo express and secrete TGF-β1 irrespective of whether they are cultured alone or co-cultured with Jurkat T cells (**Figures 3A, C**), suggesting these CRC cells have an immunosuppressive effect on the TME. MTE treatment obviously decreased the expression and secretion of TGF-β1 alleviating this effect.

Jurkat T cells were also affected by CRC cells and MTE treatment in the co-culture system. Co-culture with HCT116 or LoVo cells up-regulated FOXP3 expression in Jurkat cells (**Figure 3B**), indicating a trend to differentiate into T_reg_, and MTE treatment rescued this effect. Co-culture of Jurkat cells with HCT116 or LoVo cells resulted in increased secretion of IL-10 and decreased secretion of IL-2, which was reversed by MTE treatment (**Figure 3C**). Furthermore, PD-1 expression was not affected by co-culture or MTE treatment (**Figure 3B**). These *in vitro* results imply that MTE upregulates the immune response and reverses immunosuppression in Jurkat T cells. The western blotting results are shown in **Figure S3**.

### 3.4 MTE regulates tumor microenvironment and inhibites tumor growth in CAC and subcutaneous tumor model

**Figure 4:**
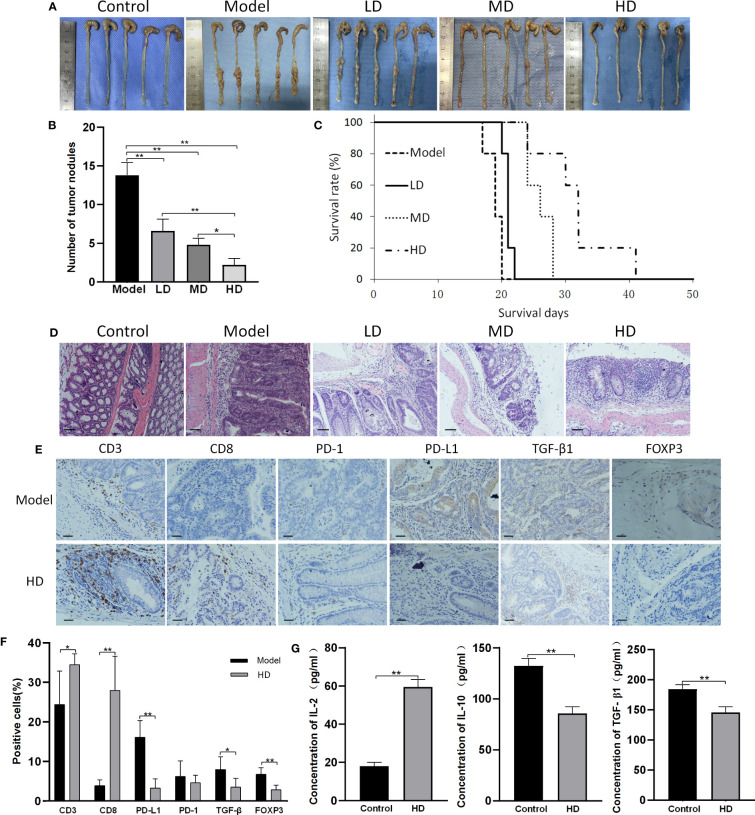
MTE treatment of CAC mice. CAC mice were generated by AOM/DSS treatment. ‘Control’ represents healthy mice that received saline but not AOM, and drank water but not DSS. ‘Model’ represents AOM/DSS treated mice. ‘LD’, ‘MD’ and ‘HD’ represent low (5 ml/kg), medium (10 ml/kg) and high (20 ml/kg) dose of MTE treatment, respectively. Scale bars, 100 μm. Data in the bar graphs are the mean ± S.D. **(A)** Images showing tumor nodules in the large intestines of CAC mice. The different groups of mice were compared for the **(B)** number of tumor nodules, **(C)** survival (survival days on the X-axis represents the time after dosing), and **(D)** H&E staining of the colons **(E)** Immunohistochemistry findings from the tumor nodules. The top row indicates the measured proteins. **(F)** Quantitation of cells with positive immunohistochemical staining. **(G)** Serum levels of IL-2, IL-10 and TGF-β1 measured by ELISA. Scale bar: 100 μm. The bar graphs present data as mean ± S.D. *P < 0.05, **P < 0.01.

As shown in **Figures 4A–C**, all colons of AOM/DSS-treated mice exhibited abundant tumor nodules, indicating the induction of CAC in these mice. MTE treatment decreased the number of tumor nodules and prolonged survival in a dose-dependent manner. Hematoxylin and eosin (H&E) staining showed that the bowel wall in the ‘Model’ group became thicker than in the ‘Control’ group (**Figure 4D**). The ‘Model’ group had severely damaged mucosa, epithelium, and crypt structure. The crypt structure and goblet cells had almost disappeared. Treatment with MTE alleviated these pathological changes. Immunohistochemistry revealed an increase in the number of tumor-infiltrating CD3^+^ and CD8^+^ T cells after MTE treatment (**Figures 4E, F**). MTE did not affect PD-1 expression in the TILs, but decreased it in the cancer cells. TGF-β1 expression in the intercellular space of the tumor and in the serum decreased following MTE treatment, indicating an inhibition of the immune escape of tumor cells in CAC mice. MTE treatment reduced FOXP3 expression in the TILs, inhibiting Treg differentiation in the tumor microenvironment in CAC mice. In addition, it increased the serum levels of IL-2, while decreasing IL-10 and TGF-β1 levels (**Figure 4G**) suggesting that MTE treatment decreases immune tolerance and promotes T cell proliferation. H&E staining and immunohistochemistry images form the other four mice are shown in **Figures S4**, **S5**, respectively.

**Figure 5:**
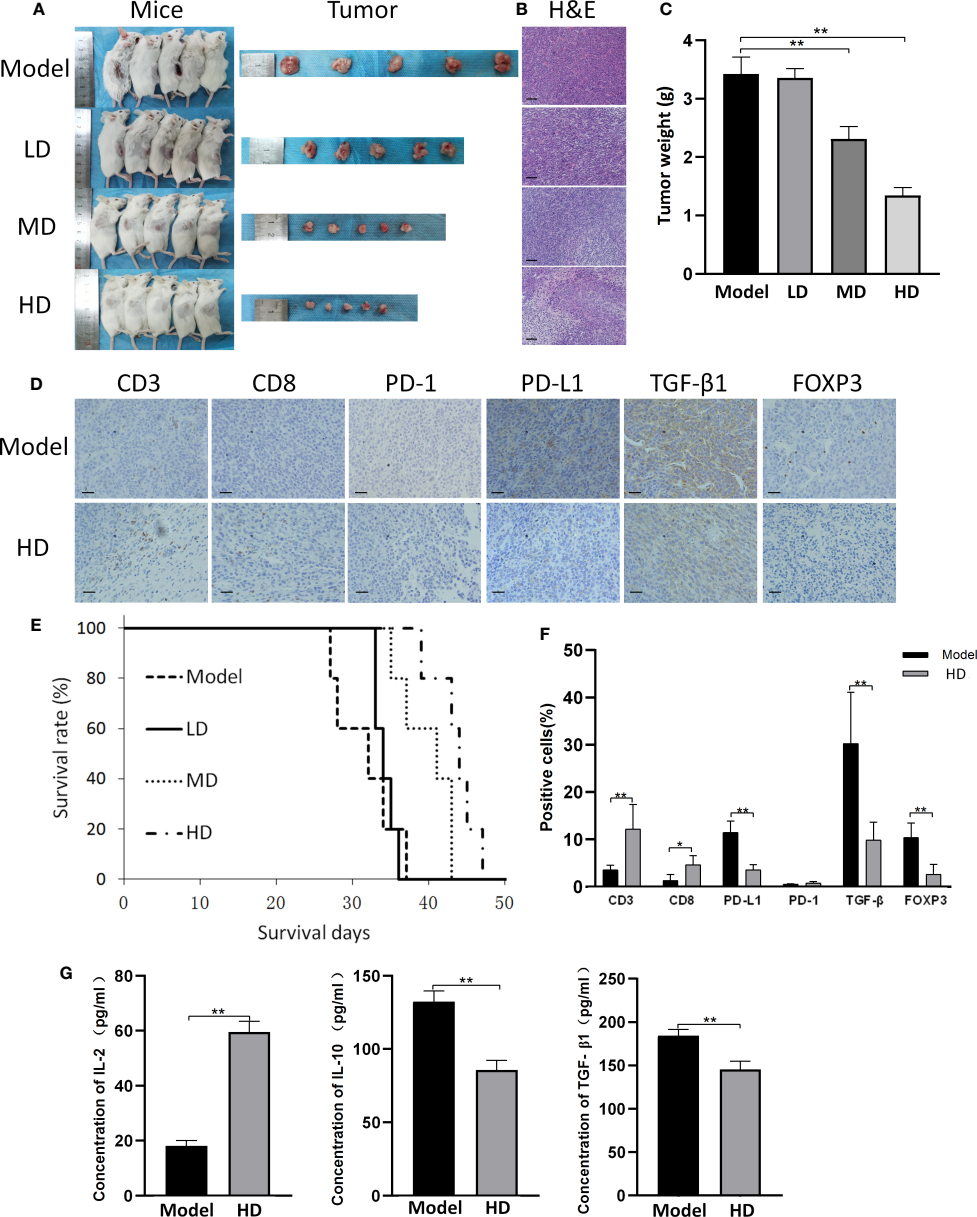
CT26 subcutaneous tumor model. ‘Model’ represents mice with subcutaneous tumors. ‘LD’, ‘MD’ and ‘HD’ represent mice treated with low (5 ml/kg), medium (10 ml/kg) and high (20 ml/kg) dose of MTE, respectively. **(A)** Shown are the tumor bearing mice before they were sacrificed and the tumors. Mice in different groups were compared for **(B)** H&E staining of the tumors and **(C)** tumor weight. **(D)** Tumors in the model and HD groups were compared for the expression of different proteins by immunohistochemistry. **(E)** Survival in the different groups. **(F)** Quantitation of cells with positive immunohistochemical staining. **(G)** Serum levels of IL-2, IL-10 and TGF-β1 measured by ELISA. Scale bar: 100 μm. The bar graphs present data as mean ± S.D. *P < 0.05, **P < 0.01.

The subcutaneous tumor model of CT26 cells exhibited results similar to those of CAC mice (**Figure 5**). Briefly, MTE treatment resulted in reduced tumor weight and increased survival time (**Figures 5A, C, E**). H&E staining of tumor samples showed the presence of high density cells with a large proportion of nucleus to cytoplasm in the ‘Model’ tumor, and an increase in the intercellular gap following MTE treatment leading to nuclear condensation (**Figure 5B**). Flaky necrotic tumor cells were seen in mice that received high dose MTE treatment (‘HD’). The changes in (a) CD8, PD-1, PD-L1, TGF-β1 and FOXP3 levels in the tumor nodules (**Figures 5D, F**) and (b) serum levels of IL-2, IL-10 and TGF-β1 (**Figure 5G**) in the MTE-treated mice were similar to those in the AOM/DSS mice, as seen by immunohistochemistry. H&E staining and immunohistochemical images of the other four mice are shown in **Figures S6**, **S7**, respectively.

**Figure 6:**
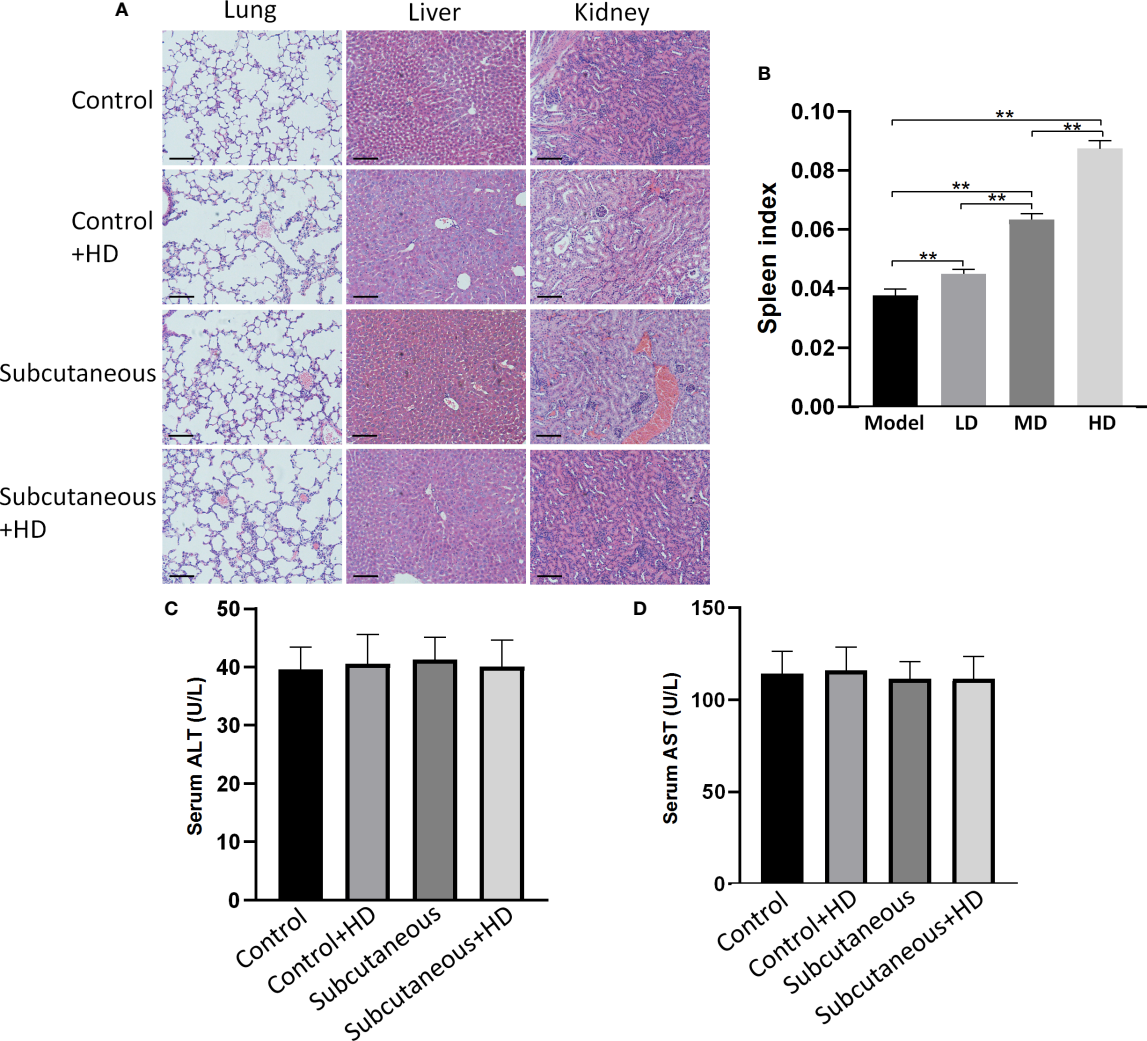
MTE does not induce toxicity in other organs. Mice were treated with low (LD, 5 ml/kg), medium (MD, 10 ml/kg), or high (HD, 20 ml/kg) dose MTE. ‘Control’ represents healthy mice, and ‘subcutaneous’ represents mice with subcutaneous tumors. **(A)** H&E staining of the lungs, liver and kidney after MTE treatment. **(B)** Changes of spleen index after different doses of MTE treatment. The mice in the different groups were compared for serum levels of **(C)** ALT and **(D)** AST. Scale bars, 100 μm. Data of columns are shown as the mean ± S.D. **P < 0.01.

MTE-treatment caused almost no toxicity in the lungs, liver, and kidneys of healthy and subcutaneous tumor model mice (**Figure 6**). It increased the spleen index in a dose-dependent manner, suggesting that an increased immune function in the treated mice. AOM/DSS treatment led to considerable toxicity in the viscus; therefore, these indices were not analyzed in the CAC mice. H&E-stained images of the other four mice are shown in **Figure S8**.

## 4 Discussion

The CRC patients in this study had stage II/III disease and were eligible for surgery. The administration of anti-tumor drugs before surgery can reduce the size of the primary tumor, increasing the feasibility and safety of the operation. Although MTE has been reported to reduce the size of primary tumors (4, 17), it remains unclear whether it regulates the tumor microenvironment. Therefore, we collected tumor tissues before and after MTE therapy to analyze the changes in the tumor microenvironment. Immunohistochemistry showed an increase in the number of CD3+/CD8+ tumor-infiltrating T lymphocytes in most patients, suggesting that MTE increases the anti-tumor T-cell immune response. CD3 is a highly specific pan-T-cell marker, expressed in all developmental stages of T cells, including CTLs, Tregs, and Th cells. In most instances, CD8 is expressed on the CRC CTLs, and serves as a specific CTL marker. Micrometastases and occult tumor cells can be detected in blood, bone marrow, and lymph nodes even in early-stage colorectal tumors. Systemic CTLs may therefore be exposed to disseminated tumor cells. Analysis of *in situ* CTLs could provide information concerning the existing cytotoxic capacity and the ability of the immune system to respond faster on tumor cell re-exposure or maintain an equilibrium state with cancer (18). Increase in CD3 and CD8 tumor-infiltrating T lymphocytes indicate a markedly better prognosis in CRC patients (18). Down-regulation of PD-L1 and TGF-β1 and FOXP3 up-regulation suggested that MTE inhibits the immune escape of tumor cells and Treg differentiation. Our findings suggest that MTE may reduce the chances of recurrence and drug resistance after surgery by improving the TME.

To further investigate the effects of MTE on T lymphocytes, we evaluated T-cell function by measuring cytokine levels in the supernatants of MTE-treated cells using a Human XL Cytokine Array. We found that MTE upregulated the immune function of Jurkat T cells, inhibited their differentiation into Tregs, and downregulated immune suppression in the tumor microenvironment. These results were consistent with the immunohistochemical findings in CRC patients, and suggested MTE may positively regulate the immunological functions of T lymphocytes. Therefore, further *in vitro* and *in vivo* experiments were performed to evaluate the effects of MTE on the tumor microenvironment.

Co-culture systems are typically used to analyze interactions between cancer and immune cells (19). These systems avoid direct contact between the two cell types and can be used to analyze the interaction between them through paracrine pathways. Our results indicated that MTE inhibited the immune escape of cancer cells and Treg differentiation of Jurkat T cells in single culture and co-culture systems. Co-culture of Jurkat T cells with cancer cells enhanced Treg differentiation, which was inhibited by MTE treatment. TGF-β1 secretion induces Treg differentiation (20), and T cells in an immunotolerant state show increased TGF-β1 pathway activity (21). Therefore, MTE may inhibit Treg differentiation by downregulating TGF-β1 secretion from cancer cells. IL-2, an approved immunotherapeutic agent for some types of cancer (22), is produced by activated CD8+ T lymphocytes and promotes the proliferation of T and B lymphocytes. It is also associated with improved disease-free and overall survival (23). Our results showed reduced IL-2 secretion in co-cultures of Jurkat and cancer cells, which was rescued upon MTE treatment. IL-10, an immunosuppressive cytokine secreted by Tregs and some cancer cells, is associated with poor prognosis in many cancers (24). MTE treatment decreased IL-10 levels in Jurkat cell supernatants both in single- and co-cultures, suggesting that MTE reduced the immunosuppressive effects of IL-10 on the tumor microenvironment. Consistent with previous studies (25), we found low MTE-induced cytotoxicity *in vitro* with high IC50 values against CRC cells. On the other hand, MTE exhibited immunoregulatory effects, resulting in the upregulation of T-cell function and inhibition of immune escape of CRC cells. These *in vitro* effects are consistent our findings in patients.

In contrast to its effects *in vitro*, MTE significantly inhibited tumor growth *in vivo* both in the CAC and subcutaneous tumor model, due to its immunoregulatory effects. The immunomodulatory effects of MTE in the two tumor models was comparable to those in the CRC patients.

TGF-β1 produced by the cancer cells and stored in the tumor microenvironment, is one of the key factors involved in their immune escape. This polypeptide cytokine impairs numerous functions of effector T lymphocytes and promotes the development and stability of FOXP3+ Tregs (26). TGF-β1-rich tumor microenvironment limits the ability of CD8+ effector T cells to eliminate tumors (27). It also cause resistance in addition to the regulation of tumor-infiltrating T lymphocytes. TGF-β1 regulates the cancer stem cell population, increasing resistance to immunosurveillanceof triple negative breast cancer (28). Our study showed that MTE reduced TGF-β1 secretion from cancer cells and FOXP3 expression in T lymphocytes *in vitro* and *in vivo*, impairing the immune escape ability of tumor cells.

Immune suppression in the tumor microenvironment increases drug resistance in cancer. For example, oxaliplatin resistance is a challenge in CRC chemotherapy. TGF-β1 induces an immunosuppressive tumor microenvironment that hinders the immune activation of oxaliplatin. TGF-β1 blockade reverses the immunosuppressive state to maintain oxaliplatin-induced immune response (29). Tregs prevent the development of effective anti-tumor immunity in patients with established tumors and promote tumor progression through their immunosuppressive roles (30). PD-L1 blockade leads to a significant increase in CD8+ lymphocytes, a significant decrease in Tregs in the tumor microenvironment, and decreased oxaliplatin resistance (31). While TGF-β1 and PD-L1 were both down-regulated by MTE, PD-1 was not affected *in vitro* and *in vivo*. Down regulation of PD-1 was also detected in a few patients after MTE therapy. Therefore, this immune checkpoint in T cells is not affected by MTE.

In conclusion, our research found that MTE enhanced the anti-tumor T-cell response both in mice and patients, and reversed the tumor microenvironment’s immunosuppressive state through inhibition of TGF-β1 secretion and PD-L1 expression in tumor cells, and inhibition of Treg differentiation. *In vitro* and *in vivo* studies demonstrated MTE’s immunoregulatory function and anti-tumor effect. The immunoregulatory function of MTE makes it suitable for application in the perioperative treatment of CRC to decrease the risk of recurrence and resistance to chemotherapy.

## Data availability statement

The original contributions presented in the study are included in the article/**Supplementary Material**, further inquiries can be directed to the corresponding author/s.

## Ethics statement

The studies involving humans were approved by the Ethics Committee of Tianjin Union Medical Center. The studies were conducted in accordance with the local legislation and institutional requirements. The human samples used in this study were acquired from a by- product of routine care or industry. Written informed consent for participation was not required from the participants or the participants’ legal guardians/next of kin in accordance with the national legislation and institutional requirements. The animal study was approved by the Animal Care and Use Committee of Tianjin Union Medical Center. The study was conducted in accordance with the local legislation and institutional requirements.

## Author contributions

BY, SZ, and YL contributed to conception and design of the study. SY organized the database. ZF, TC, JL, WW performed the statistical analysis. YW wrote the first draft of the manuscript. CZ reviewed the manuscript. All authors contributed to the article and approved the submitted version.
