# Supplementary material for: Marsdenia tenacissima enhances immune response of tumor infiltrating T lymphocytes to colorectal cancer
Source: Front Immunol. 2023 Aug 15;14:1238694. doi: 10.3389/fimmu.2023.1238694 (PMC10465246; doi:10.3389/fimmu.2023.1238694)
Supplement: Supplementary file 1 [file DataSheet_1.docx]

Supplementary Material

Marsdenia tenacissima enhances immune response of tumor infiltrating T lymphocytes to colorectal cancer

Ben Yi ^1,2†^, Shuai Zhang^2†^, Suying Yan^1,2^, Yanfei Liu^1,2^, Zhiqiang Feng^1,2^, Tianhao Chu^1,2^, Jun Liu^3^, Wei Wang^4^, Chunze Zhang^2*^, Yijia Wang^5*^

*** Correspondence:**

Chunze Zhang
chunze.zhang@nankai.edu.cn

Yijia Wang

yijiawang_1980@ nankai.edu.cn

**^†^**These authors contribute equally to this work.


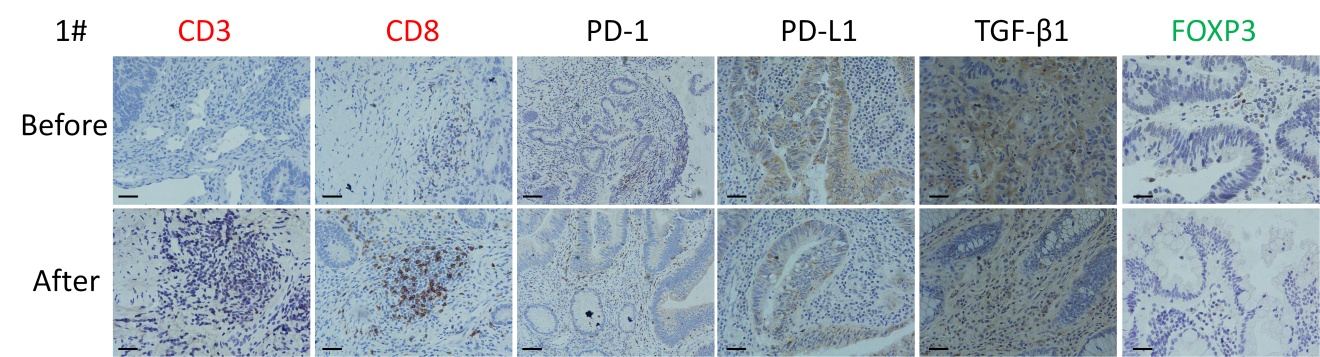


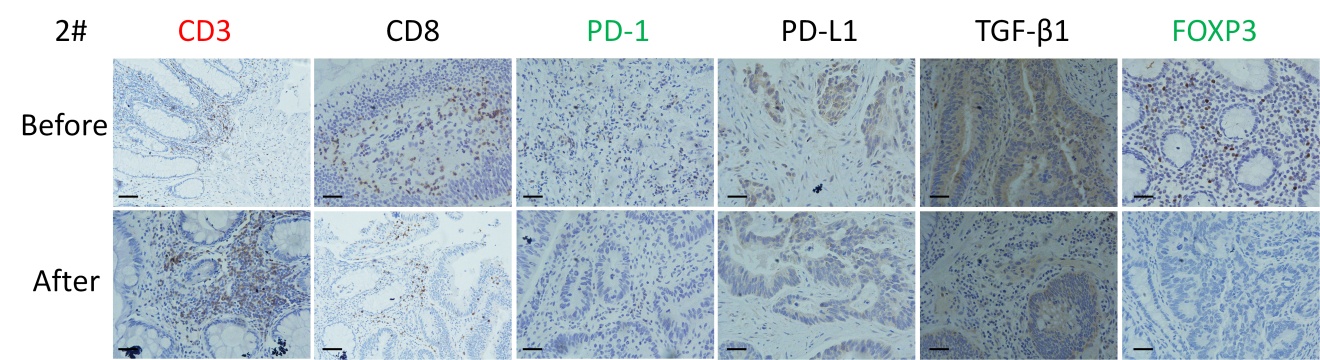


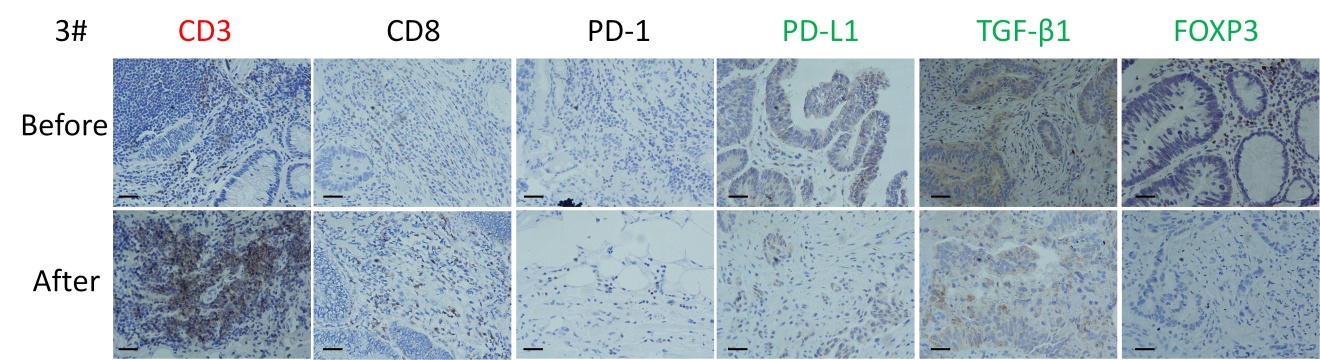


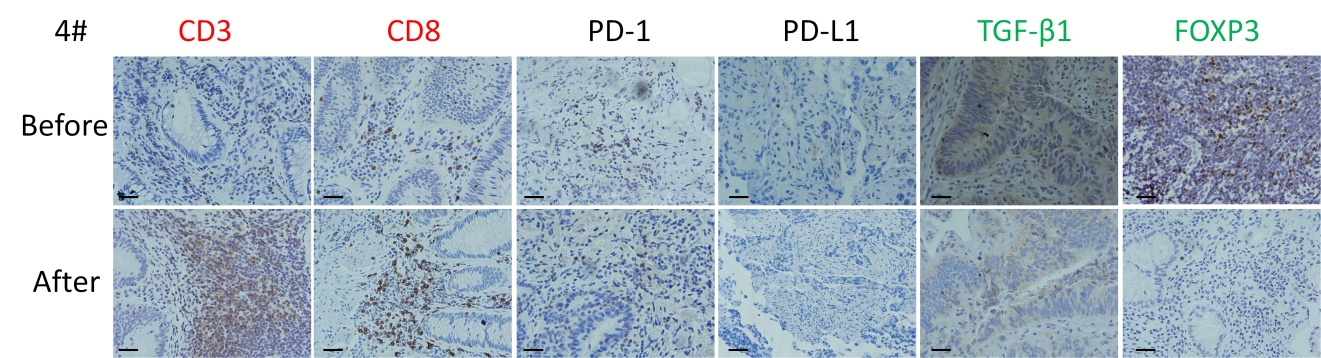


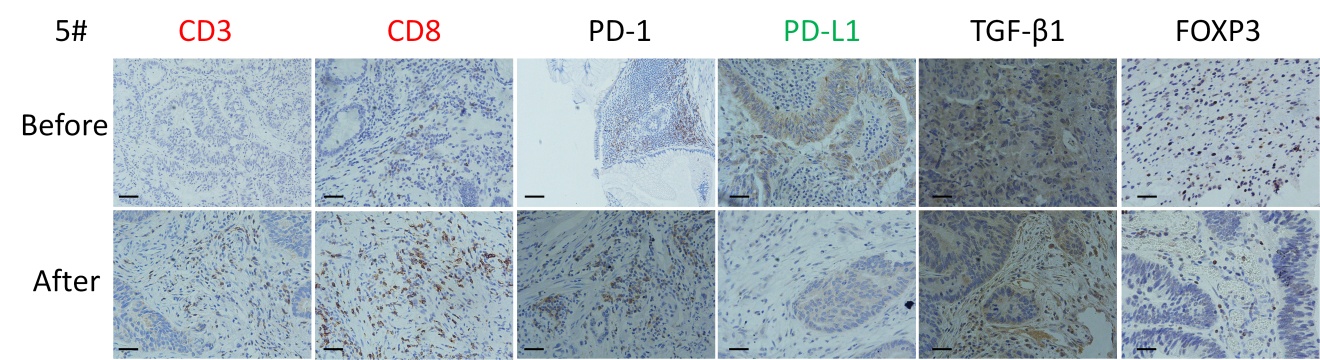


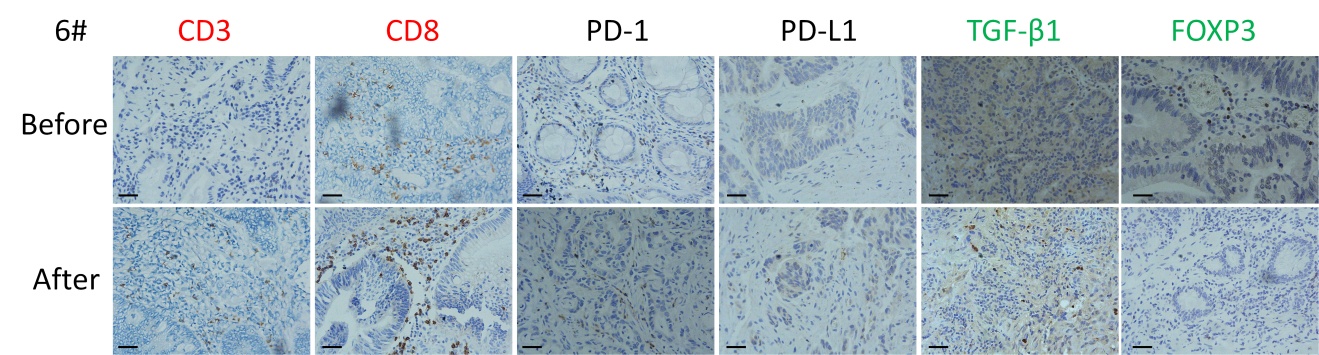


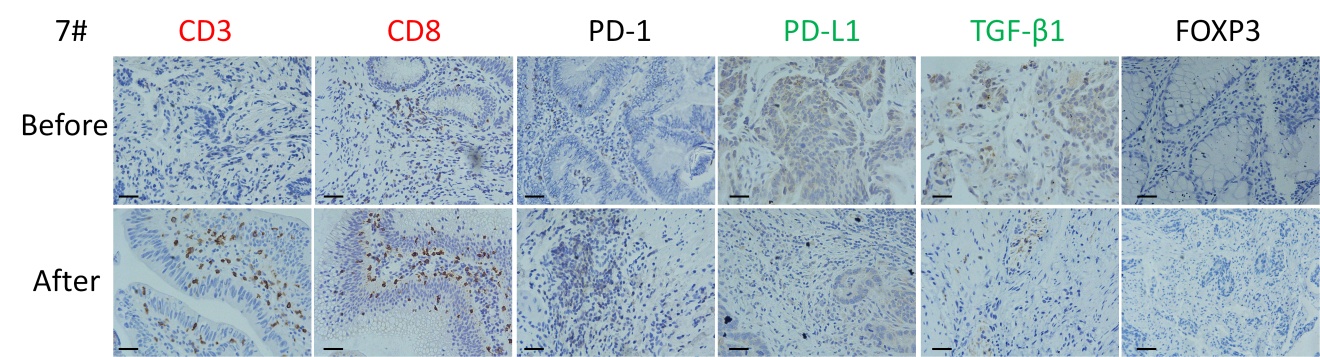


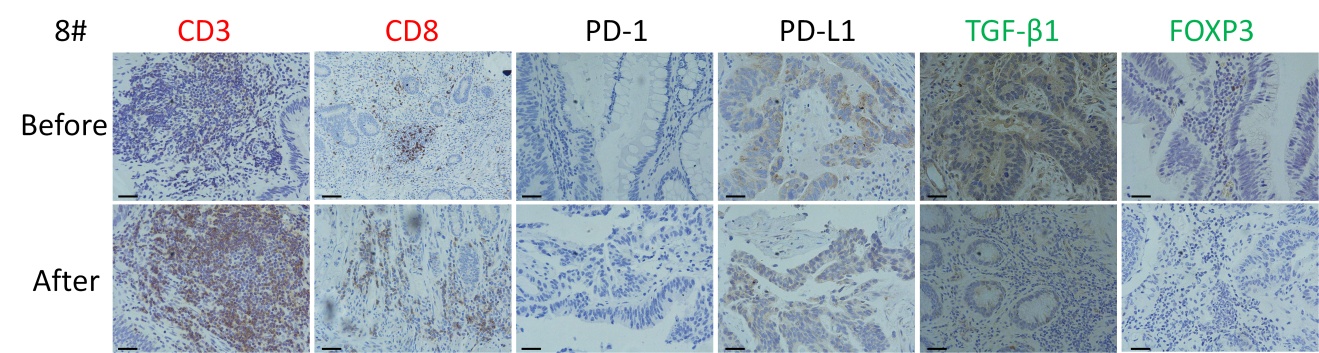


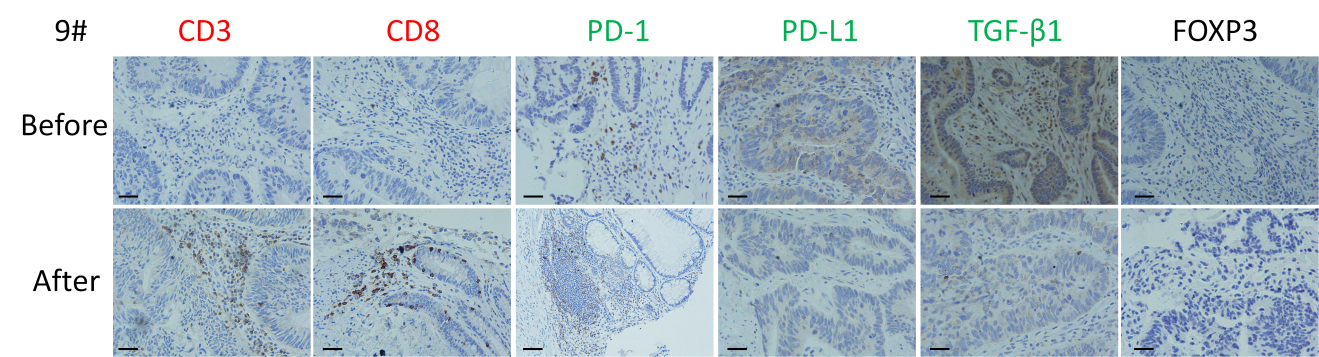


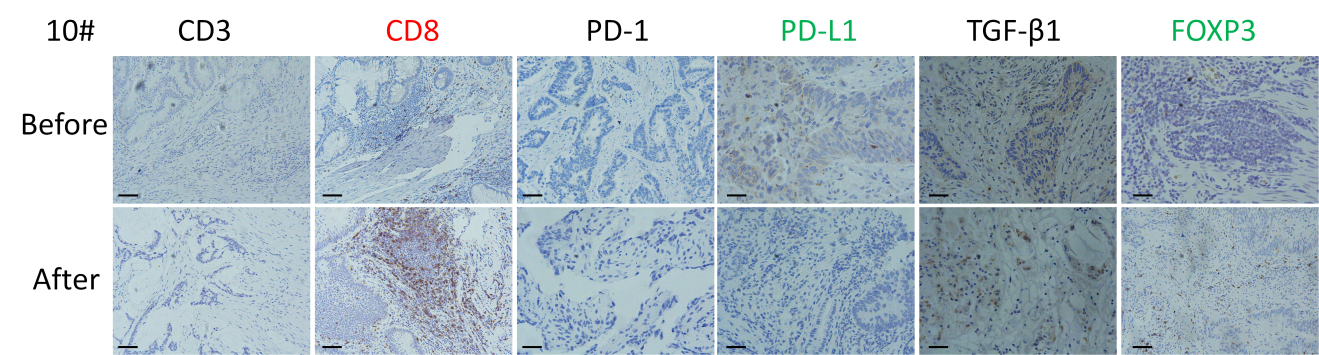


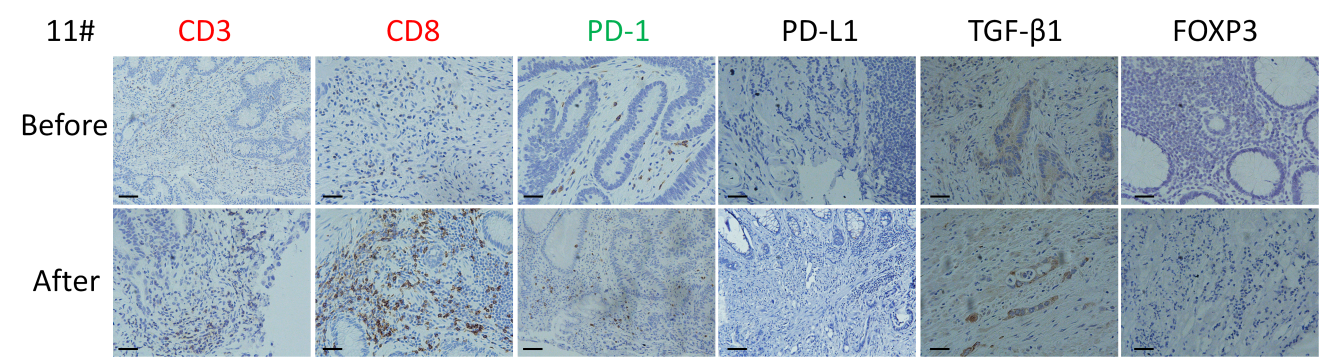


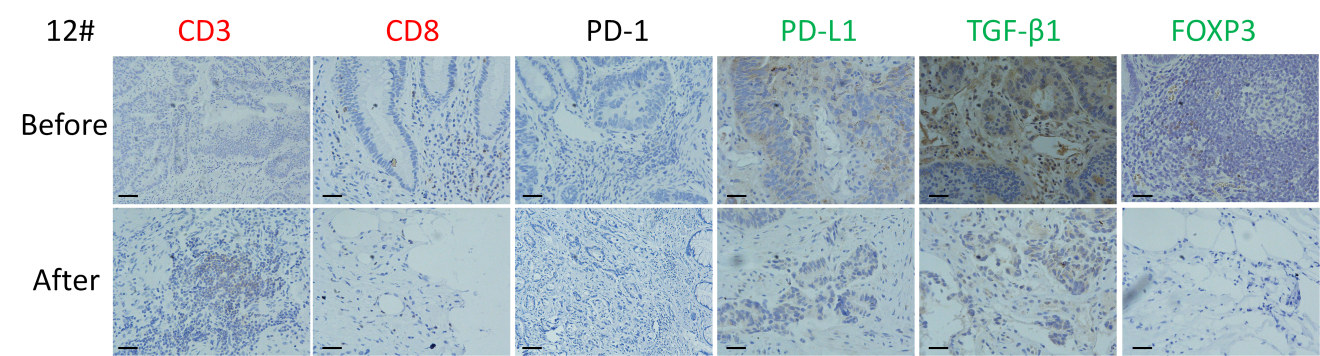


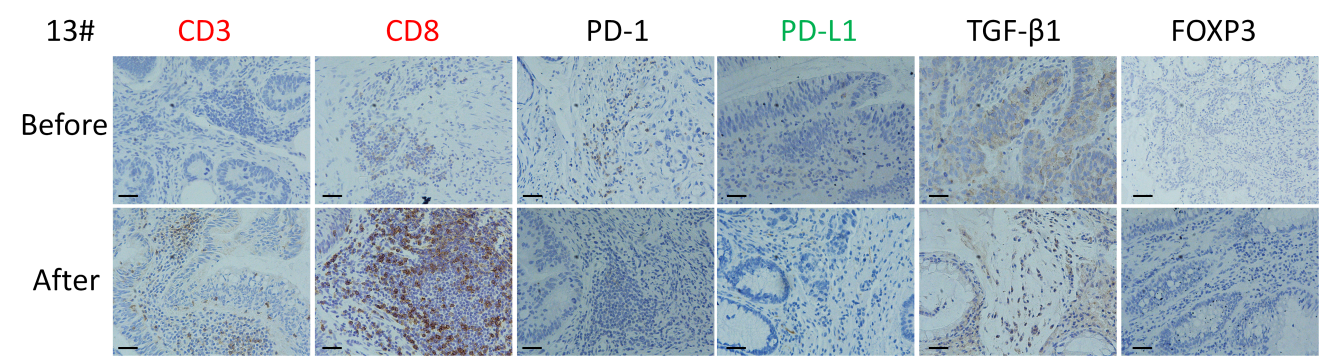


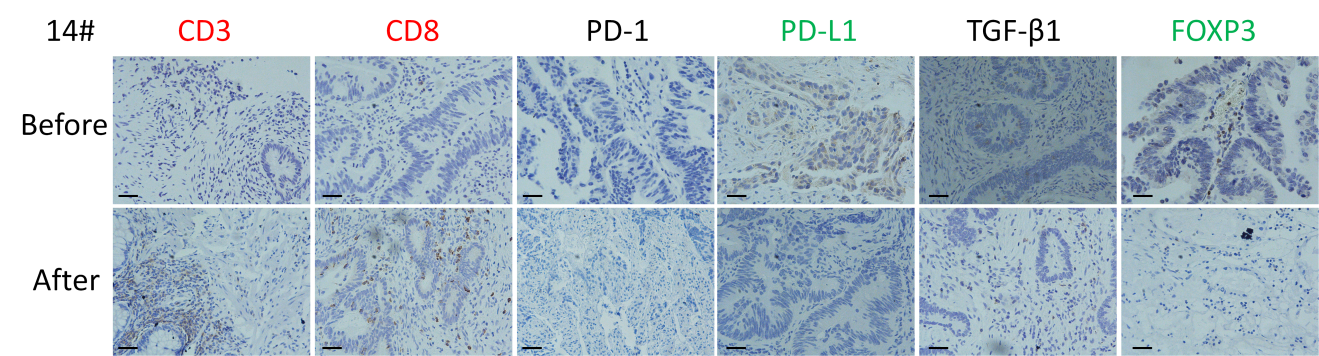


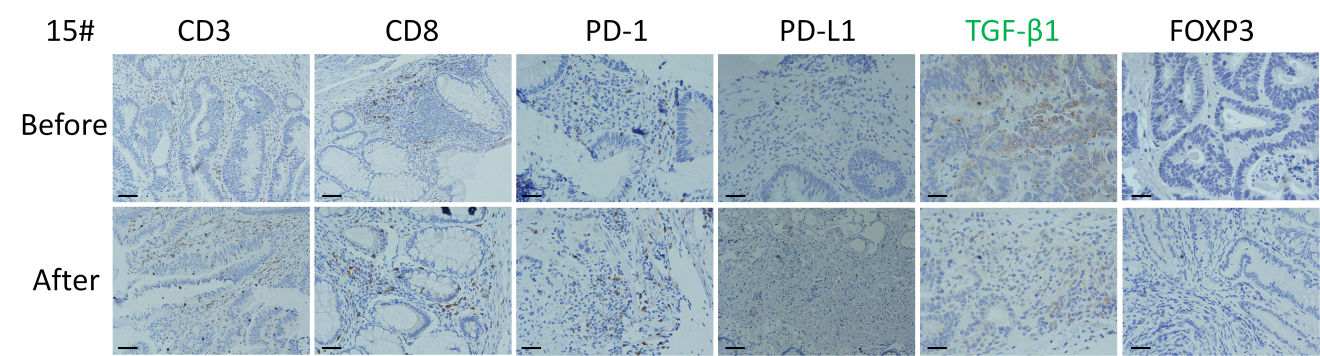


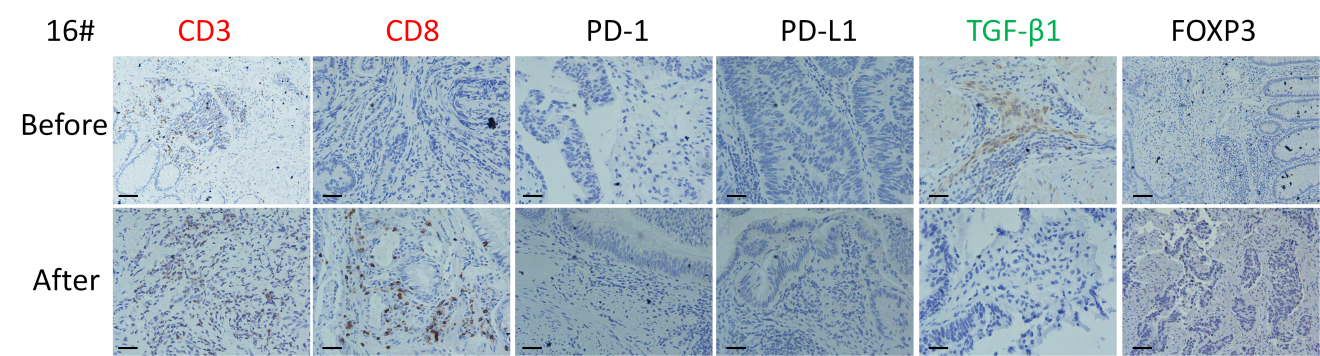


**Figure S1**. Immunohistochemistry results of 16 CRC patients. Green represents decrease, red represents increase, while black represents no change after MTE treatment.


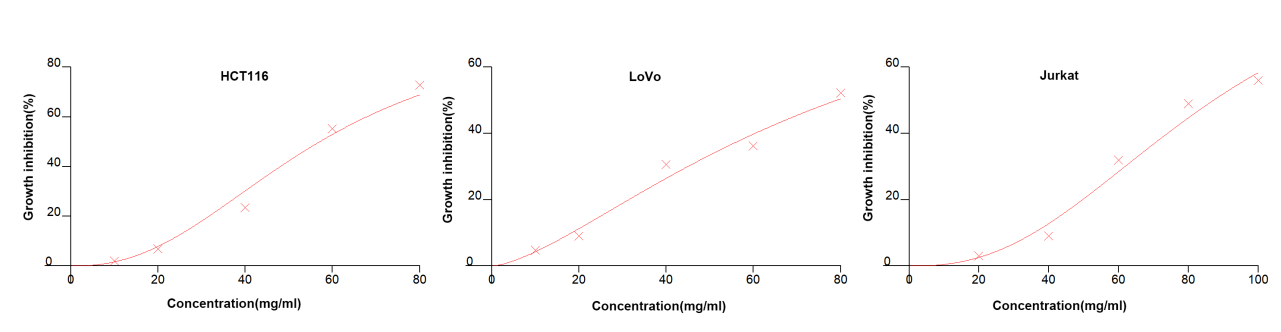


**Figure S2**. The results of cell viability assay. Cells were treated by MTE for 48 h and growth inhibition was measured by CCK-8 kit. IC50 for HCT116 is 57.11mg/ml, LoVo is 79.09mg/ml, and Jurkat T is 87.26mg/ml.


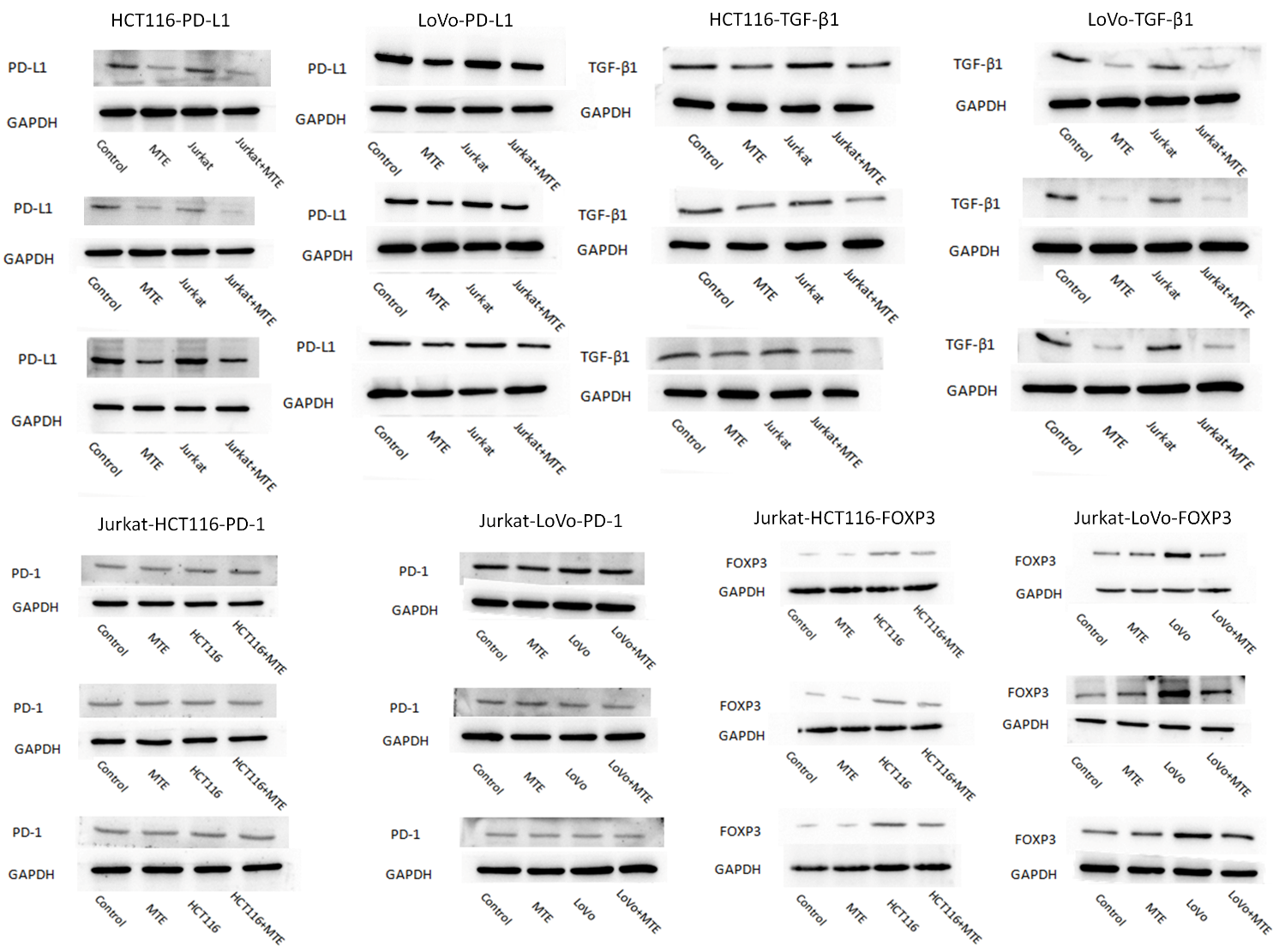





**Figure S3**. Full length blots and original images of Figure 3.


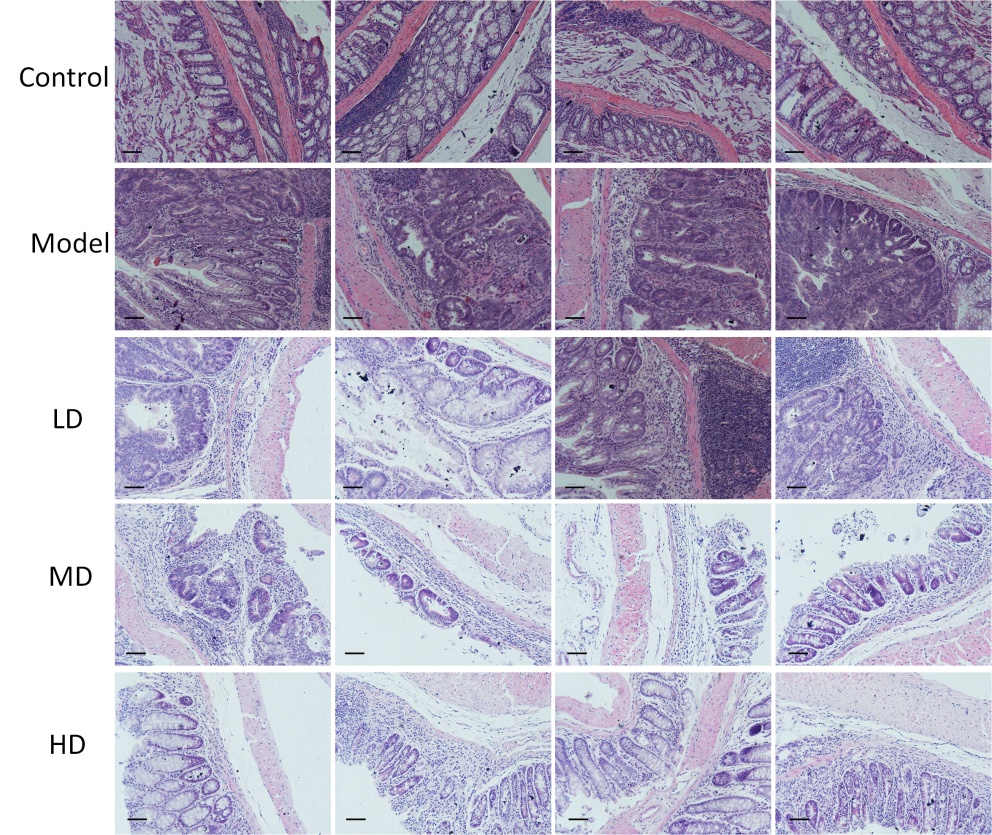


**Figure S4**. H&E stain images of colon of other 4 AOM/DSS mice. ‘Control’ represents healthy mice which were injected saline but not AOM, and drinked water but not DSS. ‘Model’ represents AOM/DSS treated mice. ‘LD’, ‘MD’ and ‘HD’ represent low (5 ml/kg), medium (10 ml/kg) and high (20 ml/kg) dose of MTE treatment, respectively. Scale bars, 100 μm.


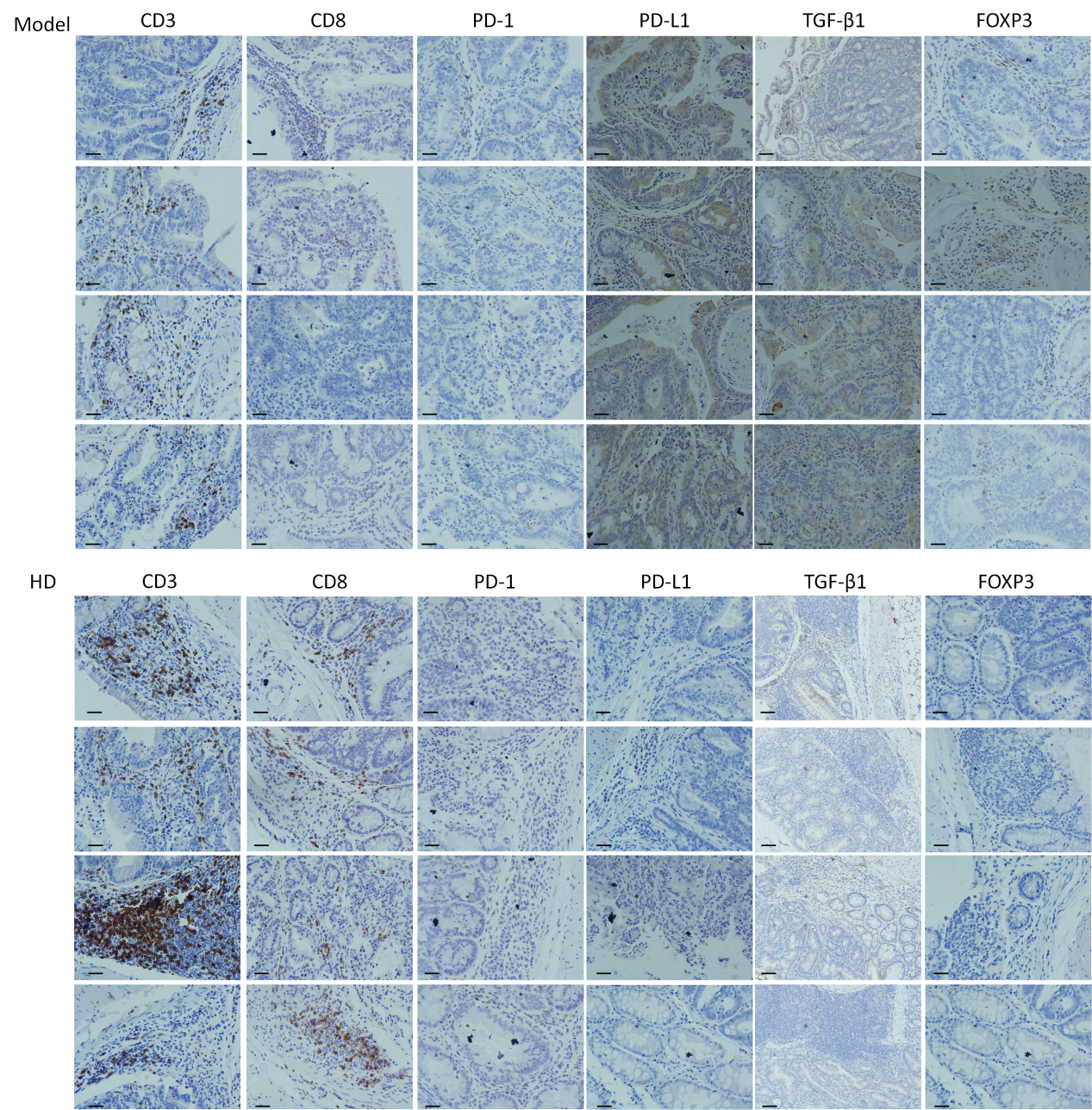


**Figure S5**. Immunohistochemistry images of tumor nodules of other 4 AOM/DSS mice. ‘Model’ represents AOM/DSS treated mice. ‘HD’ represents high (20 ml/kg) dose of MTE treatment. Scale bars, 100 μm.


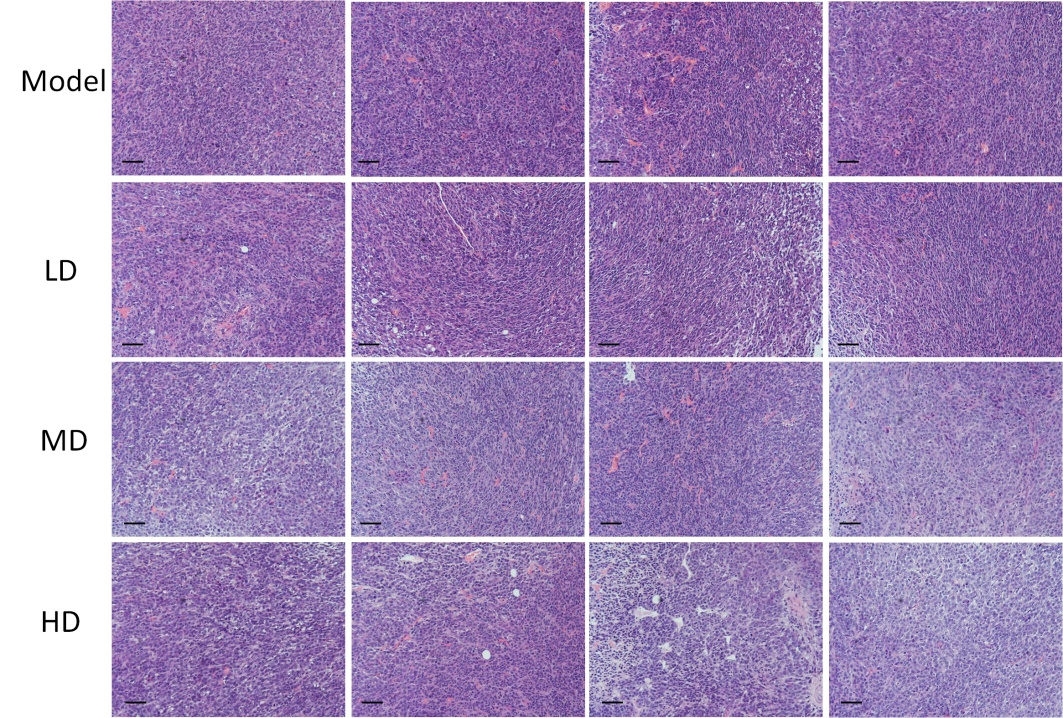


**Figure S6**. H&E stain images of tumor of other 4 subcutaneous tumor model mice. ‘Model’ represents subcutaneous tumor model. ‘LD’, ‘MD’ and ‘HD’ represent low (5 ml/kg), medium (10 ml/kg) and high (20 ml/kg) dose of MTE treatment, respectively. Scale bars, 100 μm.


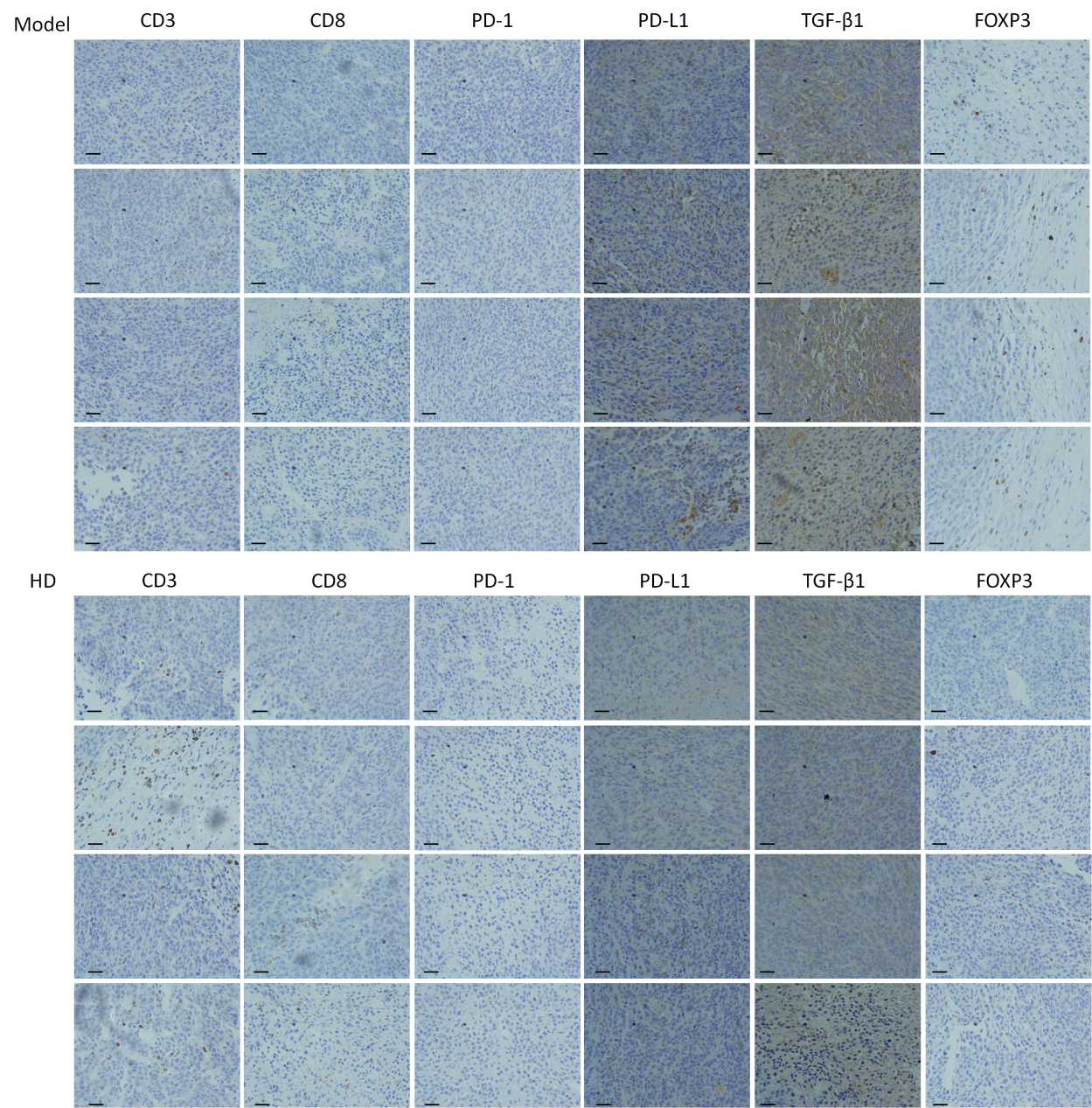


**Figure S7**. Immunohistochemistry images of tumor of other 4 subcutaneous tumor model mice. ‘Model’ represents subcutaneous tumor model. ‘HD’ represents high (20 ml/kg) dose of MTE treatment. Scale bars, 100 μm.


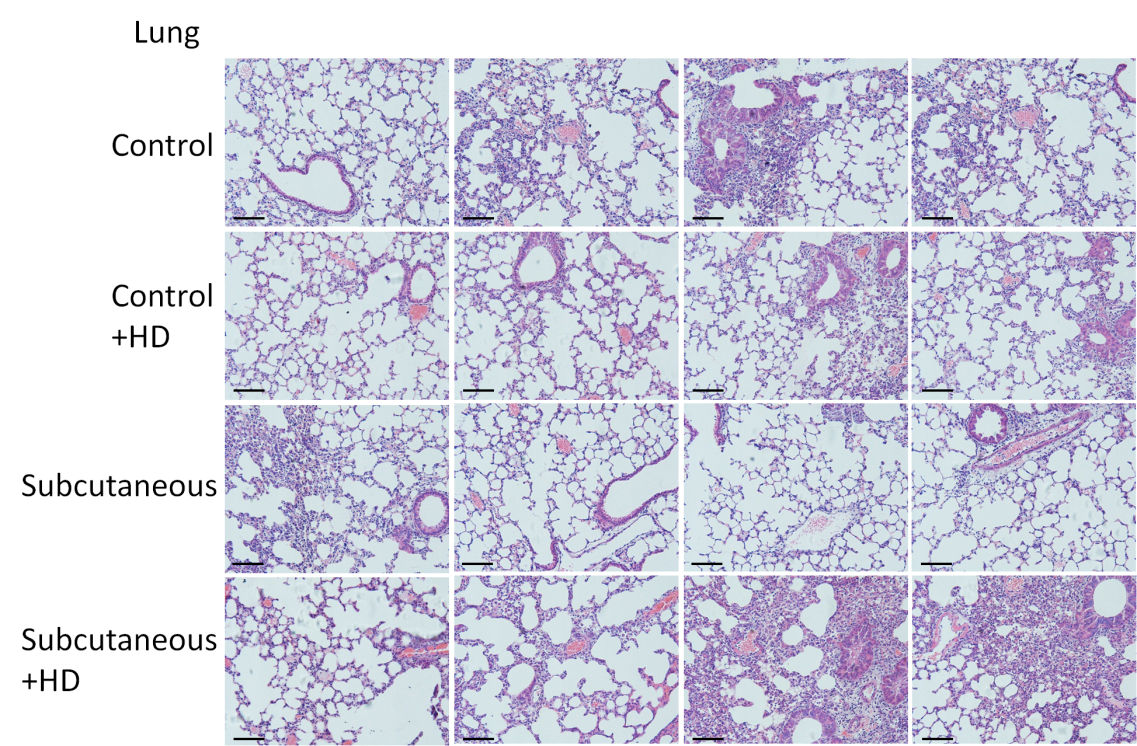


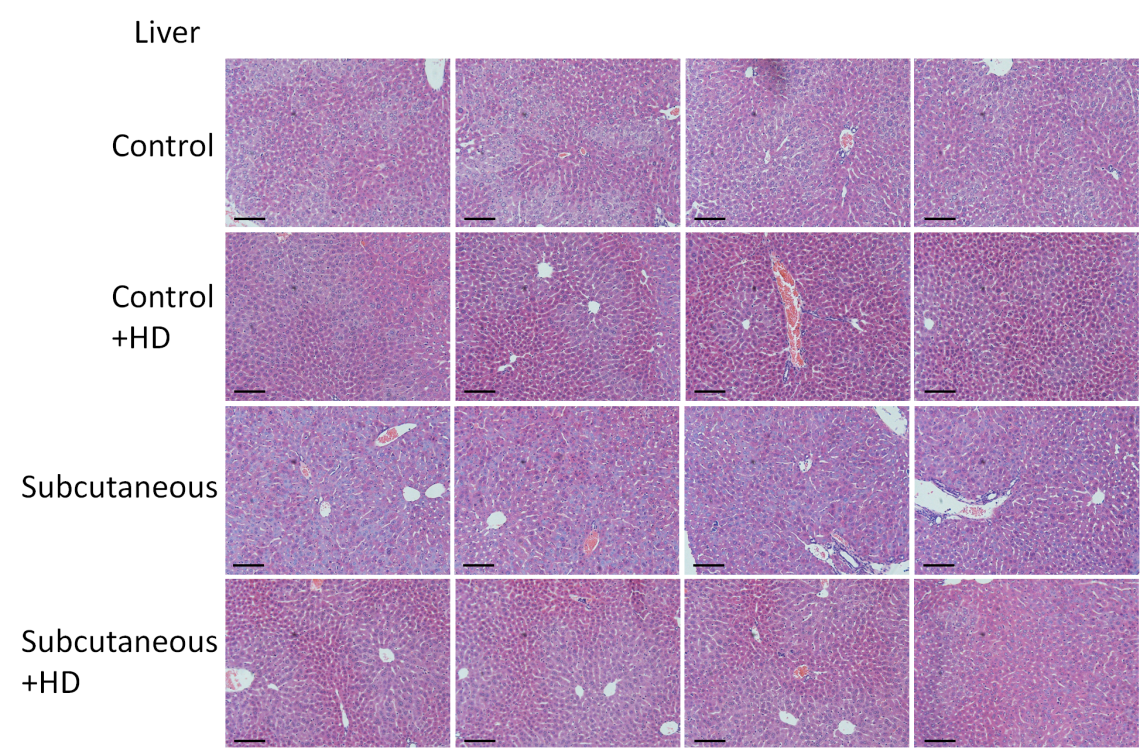


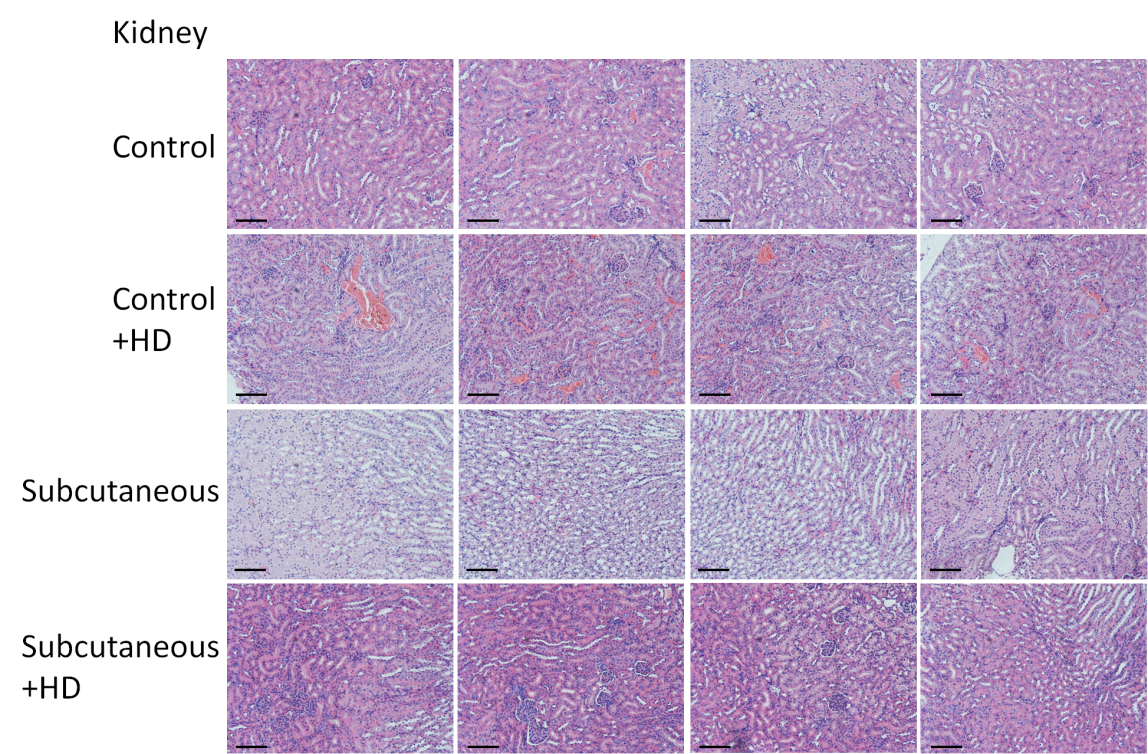


**Figure S8**. H&E images of lung, liver and kidney of other 4 mice. ‘LD’, ‘MD’ and ‘HD’ represent low (5 ml/kg), medium (10 ml/kg) and high (20 ml/kg) dose of MTE treatment, respectively. ‘Control’ represents healthy mice which were injected saline but not AOM, and drinked water but not DSS. Scale bars, 100 μm.
